# Generation and Inheritance of Targeted Mutations in Potato (*Solanum tuberosum* L.) Using the CRISPR/Cas System

**DOI:** 10.1371/journal.pone.0144591

**Published:** 2015-12-14

**Authors:** Nathaniel M. Butler, Paul A. Atkins, Daniel F. Voytas, David S. Douches

**Affiliations:** 1 Department of Plant, Soils and Microbial Sciences, Michigan State University, East Lansing, Michigan, 48824, United States of America; 2 Department of Genetics, Cell Biology and Development and Center for Genome Engineering, University of Minnesota, Minneapolis, Minnesota, 55455, United States of America; Osaka University, JAPAN

## Abstract

Genome editing using sequence-specific nucleases (SSNs) offers an alternative approach to conventional genetic engineering and an opportunity to extend the benefits of genetic engineering in agriculture. Currently available SSN platforms, such as zinc finger nucleases (ZFNs), transcription activator-like effector nucleases (TALENs), and CRISPR/Cas (clustered regularly interspaced short palindromic repeats (CRISPR)/CRISPR-associated systems (Cas)) have been used in a range of plant species for targeted mutagenesis via non-homologous end joining (NHEJ) are just beginning to be explored in crops such as potato (*Solanum tuberosum* Group Tuberosum L.). In this study, CRISPR/Cas reagents expressing one of two single-guide RNA (sgRNA) targeting the potato *ACETOLACTATE SYNTHASE1* (*StALS1*) gene were tested for inducing targeted mutations in callus and stable events of diploid and tetraploid potato using *Agrobacterium*-mediated transformation with either a conventional T-DNA or a modified geminivirus T-DNA. The percentage of primary events with targeted mutations ranged from 3–60% per transformation and from 0–29% above an expected threshold based on the number of *ALS* alleles. Primary events with targeted mutation frequencies above the expected threshold were used for mutation cloning and inheritance studies using clonal propagation and crosses or selfing. Four of the nine primary events used for mutation cloning had more than one mutation type, and eight primary events contained targeted mutations that were maintained across clonal generations. Somatic mutations were most evident in the diploid background with three of the four primary events having more than two mutation types at a single *ALS* locus. Conversely, in the tetraploid background, four of the five candidates carried only one mutation type. Single targeted mutations were inherited through the germline of both diploid and tetraploid primary events with transmission percentages ranging from 87–100%. This demonstration of CRISPR/Cas in potato extends the range of plant species modified using CRISPR/Cas and provides a framework for future studies.

## Introduction

Genome editing using sequence-specific nucleases (SSNs) is rapidly being developed as a tool for genetic engineering in crop species. Genetic engineering has played an important role in the development of modern agriculture and has contributed significantly to improvements in crop yield, quality and disease resistance [1]. Conventional genetic engineering relies on the action of trans-, intra-, or cisgenes to confer novel traits [2]. In contrast, genome editing relies on the action of SSNs to induce double-strand breaks (DSBs) at specified genomic sites and employing DNA repair pathways to incorporate target mutations through non-homologous end joining (NHEJ) or new sequence through homologous recombination (HR) [3]. Modifications are typically unlinked to integrated SSN reagents and can be segregated out of progeny. Founding SSN platforms based on natural endonucleases, such as meganucleases, have limited sequence specificity, are costly to engineer, and have limited applications for genome editing [4,5]. Subsequent SSN platforms, including zinc finger nucleases (ZFNs) and transcription activator-like effector nucleases (TALENs) are synthetic endonucleases employing a customizable DNA binding domain and the FokI nuclease [6,7]. The fusion of these domains provide flexible sequence specificity and both ZFNs and TALENs have demonstrated efficacy in a range of crop species [8–11].

CRISPR/Cas (clustered regularly interspaced short palindromic repeats (CRISPR)/CRISPR-associated systems (Cas)) represents an alternative class of SSNs that are RNA-guided endonucleases (RGENs) and feature robust activity and simple design [12]. In contrast to other SSN platforms, RGENs consist of a common nuclease and specific guide RNA to direct nuclease binding and cleavage of target DNA. The type II CRISPR/Cas system from *Streptococcus pyrogenes* used for genome editing employs a common nuclease, Cas9 and a CRISPR RNA (crRNA) and trans-activation CRISPR RNA (tracrRNA) duplex as a specific guide RNA to target protospacer-adjacent motif (PAM)-containing DNA [13]. Alone, Cas9 will bind transiently to PAM-containing DNA but requires involvement of the crRNA:tracrRNA duplex for high fidelity binding and cleavage [14,15]. For simplicity, the crRNA and tracrRNA have been fused into a single-guide RNA (sgRNA) which can be designed to target a specific sequence by modulating the first 20 nucleotides of the sgRNA to match the complementary strand of a ‘protospacer’ target DNA site [13]. Co-expression Cas9 with one or more sgRNA provides a two-component system capable of targeting multiple loci for modification [16].

Genetic engineering in agriculture is at an important crossroads. Increasing pressure from the public to develop ‘safer’ biotechnology and advances in SSN and next-generation sequencing (NGS) technology have provided an opportunity to advance genome editing methodology and usher in a new generation of genetic engineering [17]. Most crops, like potato (*Solanum tuberosum* Group Tuberosum L.) are amendable to plant transformation but lack sufficient genetic resources to validate genetic studies and assess gene function. Random mutagenesis using ethyl methanesulfonate (EMS), radiation, or T-DNA integration requires generating large mutant collections and extensive screening to identify informative mutants. CRISPR/Cas and other SSN platforms allow mutagenesis of target genes and direct assessment of gene function.

This report demonstrates the use of CRISPR/Cas for targeted mutagenesis in both diploid and tetraploid potato. CRISPR/Cas reagents targeting the potato *ACETOLACTATE SYNTHASE1* (*StALS1*) gene were expressed in leaf explants via *Agrobacterium tumefaciens* (*Agrobacterium*) using a conventional 35S T-DNA expression vector [18] or a modified geminivirus T-DNA expression vector [19]. Both sgRNAs and T-DNAs tested were capable of generating targeted mutations in stable events. Single targeted mutations in primary events were capable of being carried through clonal generations and the germline as Cas9-free progeny.

## Materials and Methods

### Plant materials

The tetraploid *S*. *tuberosum* cultivar “Désirée” (Désirée) and a diploid self-incompatible breeding line, MSX914-10 (X914-10) were used in the study. X914-10 was produced from a cross between the doubled-monoploid (DM) *S*. *tuberosum* Group Phureja line used to construct the potato reference genome [20] and 84SD22, a heterozygous *S*. *tuberosum* x *S*. *chacoense* hybrid breeding line and has high transformation efficiency [21]. Désirée is a red-skinned variety with high transformation efficiency [22]. Three to four-week-old tissue culture plants used for *Agrobacterium* transformation were grown in Magenta^®^ boxes (Phytotech, Shawnee Mission, KS) on light racks set to 16-h-light/8-h-night photoperiod at 22C. Eight to ten-week-old soil-grown plants used in crosses or selfing were grown in greenhouses under the same photoperiod as tissue culture plants. Fruit was harvested three weeks following fruit set. An inbred diploid line, M6 [23] was used in crosses with X914-10 events while Désirée events were selfed.

### CRISPR/Cas reagent preparation

The *Streptococcus pyogenes* Cas9 gene was codon-optimized for *Arabidopsis* and synthesized (GenScript, Piscataway, NJ) as previously described [19]. Cas9 and individual sgRNA driven by the *Arabidopsis* U6 RNA pol III promoter [19] were cloned into Gateway-compatible binary vectors pMDC32 (35S; [18]) and pLSL (LSL; [19]). The Rep and RepA coding sequences from the *Bean Yellow Dwarf Virus* (*BeYDV*) were cloned into the pMDC32 vector for co-expression with pLSL reagents [19].

### *Agrobacterium*-mediated transformation

*Agrobacterium*-mediated transformation of potato leaf explants was conducted as previously described [22]. Approximately 20–40 hygromycin-resistant events rooting in 5 mg/L hygromycin B (Life Technologies, Grand Island, NY) were sampled for each transformation. Callus was sampled by excising wounded surfaces of leaf explants that included both callus and non-callus tissues. Sampled callus from three to four leaf explants were combined for genomic DNA extractions.

### Enrichment and T-DNA PCR and restriction enzyme digestion assays

Genomic DNA was extracted from callus and leaf tissues using the DNeasy Plant Mini kit (Qiagen, Valencia, CA). For T-DNA PCRs, primers 5’-CCTGTCGTGCCAGCTGC-3’ and 5’-TGTTGAGAACTCTCGACGTCCTGC-3’ were used for LSL T-DNA, and primers 5’- CGAGCTCCACCGCGG-3’ and 5’-CCTCCTTAGACGTTGCAGTC-3’ for Rep T-DNA. Primary PCR amplicons of *StALS* loci were generated using primers 5’-GGTTGACATTGATGGTGAC-3’ and 5’-GCCTAGAACTAGTTATGTAG-3’ with 100 ng genomic DNA and Phusion High-Fidelity DNA Polymerase (NEB, Ipsich, MA). Primary amplicons were purified using the QIAquick PCR purification kit (Qiagen, Valencia, CA) and digested overnight with *Alo*I (Life Technologies, Grand Island, NY) or *Bsl*I (NEB, Ipswich, MA) using recommended conditions. Resistant bands were purified from 2% agarose gels using QIAquick Gel Extraction kit (Qiagen, Valencia, CA) and subcloned using the Topo TA Cloning kit (Life Technologies, Grand Island, NY) for Sanger sequencing at the Michigan State University Research Technology Support Facility (MSU-RTSF).

## Results and Discussion

**Fig 1:**
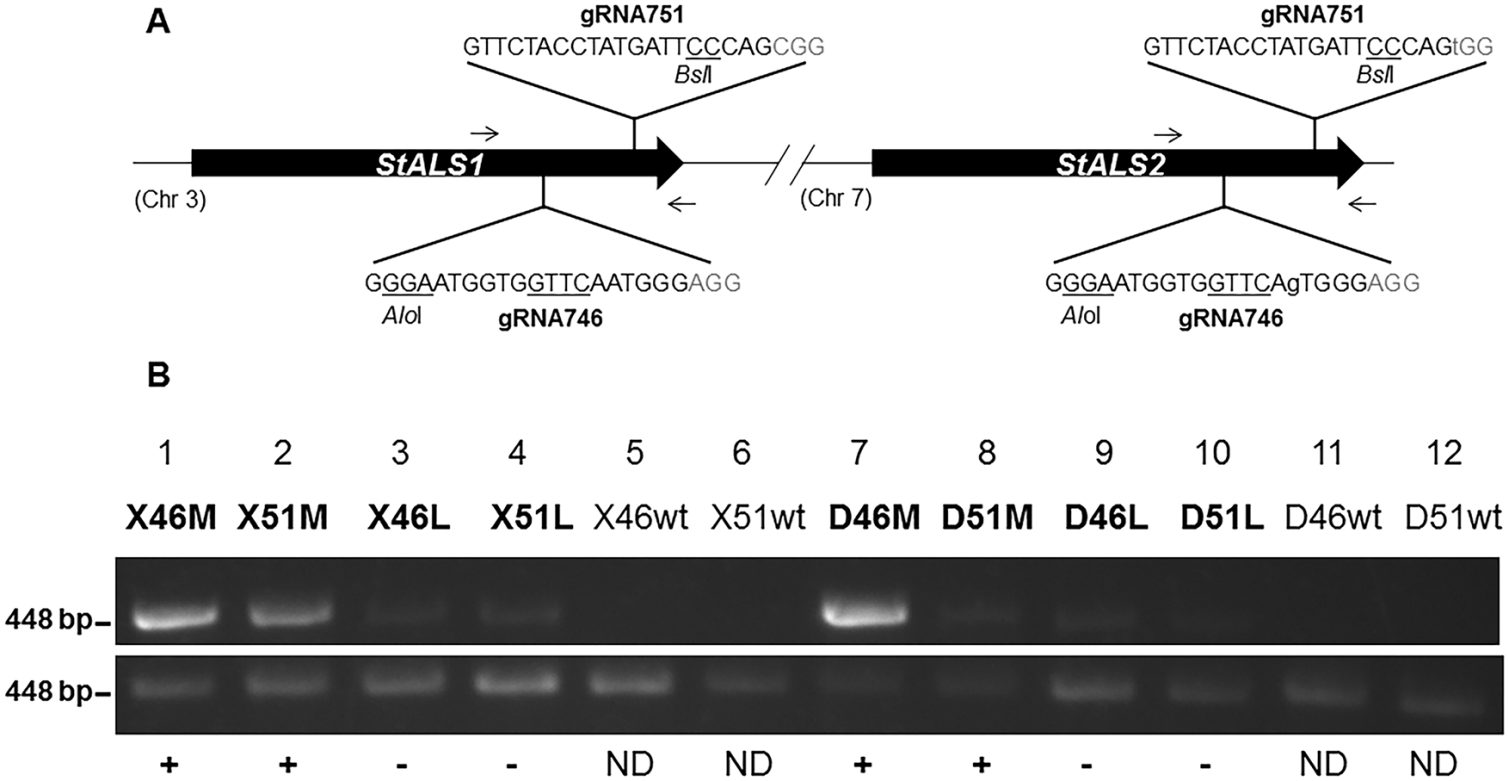
Generation of targeted mutations in callus tissues of potato using CRISPR/Cas reagents. **A**. Target sites of single-guide RNA within potato *StALS1* and -*2* genes. A single nucleotide polymorphism (lowercase) exists in the gRNA746 target site of *StALS2* but not gRNA751. *Alo*I and *Bsl*I restriction enzyme sites exist in sgRNA target sites of both genes (underlined). Arrows indicate primers used for enrichment PCR and restriction enzyme digestion assays. PAM sequences are in gray. **B**. Modified enrichment PCR assay using potato callus tissue transformed with gRNA746 and gRNA751 CRISPR/Cas reagents. Total genomic DNA was subjected to PCR amplification of the *StALS* target site (bottom image; 448 bp), digested overnight with *Alo*I (lanes 1, 3, 5, 7, 9, 11) or *Bsl*I (lanes 2, 4, 6, 8, 10, 12), and reamplified (top image; 448 bp) to generate an enriched amplicon. Enriched band intensities were normalized by dividing the quantified band intensity of the enriched band by the primary PCR amplicon (S1 Table). Positive (+), negative (-) and non-detectable (ND) enriched bands have normalized intensities equal or over 0.5, less than 0.5 and equal or more than 0.05, or less then 0.05, respectively. Diploid (X; lanes 1–6) and tetraploid (D; lanes 7–12) genotypes were tested using both sgRNAs in the conventional 35S (M; lanes 1, 2, 7, 8) and geminivirus LSL (L; lanes 3, 4, 9, 10) T-DNA backbones. Wild-type (wt; lanes 5, 6, 11, 12) genomic DNA was used as non-transformed controls.

The *StALS1* gene was chosen as a target locus for designing two sgRNAs (gRNA746 and gRNA751) due to its potential function in herbicide resistance [24]. Each sgRNA target site is separated by 215 base pairs (bp) and is localized to the 3’ end of the *StALS1* coding sequence (Fig 1A). A closely related paralog of *StALS1* (PGSC0003DMG400034102), *StALS2* (PGSC0003DMG400007078), is also targeted by gRNA751 and contains a single nucleotide polymorphism (SNP) in the target site of gRNA746 (Fig 1A; lowercase). Both sgRNAs include restriction enzyme sites proximal to the PAM to facilitate detection and cloning of NHEJ mutations at target loci (Fig 1A; underlined).

*Agrobacterium*-mediated delivery of CRISPR/Cas reagents was used for both transient expression and generation of primary events (S1 Fig). Expression of an *Arabidopsis* codon-optimized Cas9 and individual sgRNAs were driven by a doubled cauliflower mosaic virus 35S promoter and *Arabidopsis* U6 promoter, respectively, in either a conventional pCambia T-DNA backbone (35S; [18]) or modified geminivirus backbone (LSL; [19]) (S2 Fig). Geminivirus T-DNA constructs were co-transformed with a conventional T-DNA constitutively expressing the Rep/RepA (Rep) coding sequences required for geminivirus replicon release and expression of Cas9 [19].

One week following *Agrobacterium* infection, calli and surrounding tissue was collected and total genomic DNA extracted for target mutation detection using enrichment PCR (Fig 1B and S3 Fig). A conventional enrichment PCR failed to detect targeted mutations in most samples with only slight detection in Désirée calli transformed with gRNA746 in the 35S T-DNA (S3 Fig; arrows). A modified enrichment PCR achieved more sensitive detection and was used to determine if the callus samples contained targeted mutations (Table 1 and S1 Table). Overall, targeted mutations could be detected in calli of both genotypes using either sgRNA in the conventional 35S T-DNA but not the geminivirus LSL T-DNA or non-transformed tissues. The reduction of targeted mutations in calli transformed with the geminivirus LSL T-DNA was also observed in *Agrobacterium*-infiltrated tobacco leaves and supports the use of the geminivirus vector system for promoting HR rather than NHEJ mutagenesis [19].

**Table 1:** Summary of targeted mutation screen of primary events and enrichment PCR results from callus. Diploid (X914-10) and tetraploid (Désirée) genotypes were stably transformed with gRNA746 and gRNA751 CRISPR/Cas reagents in a conventional 35S or geminivirus LSL T-DNA backbone using hygromycin selection (Total events). A restriction enzyme digestion assay and quantification of resistant and digested bands were used to identify events with at least 1% mutation frequencies (# with mutations) and events above a threshold using expected single allele mutation frequencies (# above threshold) (S2 Table). Percentages are of total events and modified enrichment PCR results come from Fig 1B and S1 Table.

Genotype	gRNA	T-DNA	Total events	# with mutations	% with mutations	# above threshold	% above threshold	Modified Enrichment PCR
X914-10	746	35S	27	15	55%	4	15%	+
X914-10	746	LSL	32	13	41%	1	3%	-
X914-10	751	35S	35	3	9%	1	3%	+
X914-10	751	LSL	39	1	3%	0	0%	-
Désirée	746	35S	35	21	60%	10	29%	+
Désirée	746	LSL	33	12	36%	0	0%	-
Désirée	751	35S	37	4	11%	1	3%	+
Désirée	751	LSL	21	1	5%	0	0%	-

**Fig 2:**
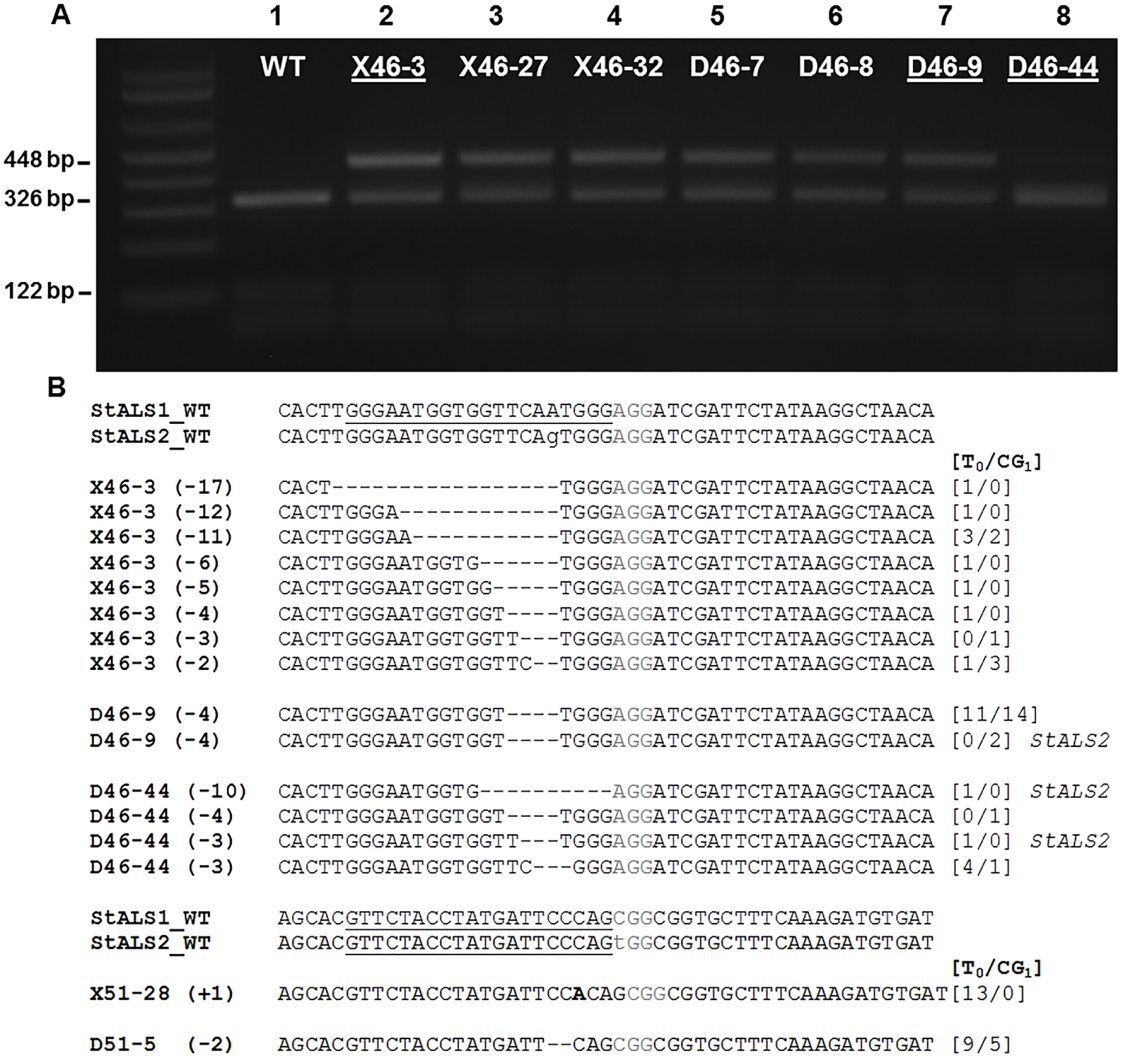
Generation and cloning of targeted mutations in primary events of potato using CRISPR/Cas reagents. **A**. Restriction enzyme digestion assay of diploid (X; lanes 2–4) and tetraploid (D; lanes 5–8) primary events. Total genomic DNA from primary events was subjected to PCR amplification of the *StALS* target site and digested overnight with *Alo*I yielding a 448 bp resistant band and 326 bp and 122 bp digested bands. Wild-type X914-10 (WT; lane 1) and Désirée (Fig 3A) genomic DNA were used as a negative controls. **B**. Cloned targeted mutations in primary events of potato. Diploid (X) and tetraploid (D) events constitutively expressing gRNA746 (46) and gRNA751 (51) CRISPR/Cas reagents were used for cloning. Resistant bands from restriction enzyme digestion assays were excised from 2.0% agarose gels, purified, and subcloned for Sanger sequencing. Sanger reads from each event were aligned to *StALS1* and -*2* wild-type sequence (WT) from each sgRNA target site (gRNA746; top alignments, gRNA751; bottom alignments). The lengths of deletions (-) or insertions (+) are in parenthesis to the left of each cloned mutation and the number of reads generated in the primary event (T_0_) or first clonal generation (CG_1_) are in brackets on the right. All targeted mutations were cloned from *StALS1* unless indicated on the right. PAM sequences are in gray.

To determine if targeted mutations detected in calli could also be detected in stable expression lines, transformed calli were regenerated and resulting primary events were screened for targeted mutations using a restriction enzyme digestion assay (Fig 2A, Table 1 and S2 Table). Hygromycin selection was used during regeneration and in a rooting assay to generate stable events (Table 1; total events). Total genomic DNA was extracted from leaf tissue of primary events (T_0_) and used for PCR amplification and overnight digestion with a restriction enzyme that cleaves within the sgRNA target site (Fig 1A). Digested amplicons were subjected to gel electrophoresis and targeted mutation frequencies estimated using the fraction of resistant band intensity divided by the sum of the resistant and digested band intensities (S2 Table). Events with targeted mutation frequencies equal or greater than a 25% and 12.5% threshold for X914-10 and Désirée, respectively were considered mutant events based on expected single allele mutation frequencies across both *StALS* loci (Table 1; # above threshold).

In X914-10, mutant events accounted for 15% (gRNA746) and 3% (gRNA751) of 35S T-DNA lines and 3% (gRNA746) and none (gRNA751) of LSL T-DNA lines. In Désirée, mutant events accounted for 29% (gRNA746) and 3% (gRNA751) of 35S T-DNA lines and none of the LSL expression lines (Table 1). An increase in the number of mutant events from gRNA746 in relation to gRNA751 using the conventional 35S T-DNA (approximately five and ten-fold for X914-10 and Désirée, respectively) may be due to a GG motif at the 3’ end of the target sequence of gRNA746 but not gRNA751 that has shown to improve gRNA efficiency (Fig 1A) [25,26]. Furthermore, the lack of mutant events from using the geminivirus T-DNA support the results of the transient assay but also could be the result of inefficient co-transformation of the geminivirus and the Rep T-DNAs. To investigate this possibility, primers specific to each T-DNA were used in a PCR assay (S4 Fig). Although the Rep T-DNA could be detected in most events, the LSL T-DNA could only be clearly detected in one event although some detection was seen in others (S4B Fig; lane 13). These results suggest the LSL T-DNA is not properly integrating into the genomes of primary events and hygromycin resistance is likely derived from integration of the Rep T-DNA in the genomes of geminivirus-modified events. Hence, the number of geminivirus-modified events with targeted mutations could be been underestimated due to selection for Rep T-DNA integration and not the LSL T-DNA carrying the CRISPR/Cas reagents.

In order to characterize mutant alleles in stable expression lines and track them across clonal generations, a subset of nine mutant events derived from the gRNA746 35S T-DNA, four from X914-10 and five from Désirée, were vegetatively propagated in tissue culture (clonal generation 1; CG_1_) using shoot tip explants and leaf tissue sampled for mutation cloning. Total genomic DNA from both clonal generations, T_0_ and CG_1_ were used in restriction enzyme digestion assays to produce resistant bands (Fig 2A). Resistant bands were excised and subcloned for Sanger sequencing. Sequence reads were aligned with wild-type sequence to identify mutant alleles and their corresponding locus (i.e. *StALS1* or -*2*) (Fig 2B and S5 Fig).

Insertion-deletion mutations were identified in all nine mutant events ranging from a single bp insertion (X51-28 (+1)) to a 38 bp deletion (X46-27 (-38)) (Fig 2B and S5 Fig). Eight of the nine events maintained a mutation type across clonal generations (X46-3, -27, -32; D46-7, -8, -9, -44; D51-5) due to the lack of targeted mutations detected in the nine sequencing clones from the CG_1_ generation of X51-28. Four of the nine mutant events had more than one mutation type and most likely contain somatic mutations (X46-3, -27, -32 and D46-44). Somatic mutations were most evident in the diploid background with three of the four events having more than two mutation types at a single locus (X46-3, -27 and -32). Conversely, in the tetraploid background, four of the five events carried only one mutation type and in two of these cases, carried the same mutation at both *StALS* loci (Des46-7 and -9). The occurrence of a 4 bp deletion across D46-7, -8 and -9 is most likely an artifact of transformation (i.e. taken from the same callus). Nevertheless, discovery of the same 4 bp deletion mutation at different loci within D46-7 and -9 suggests a preference for this mutation type and might be explained by microhomology (“TGG”) within the gRNA746 target site [27]. Furthermore, the incidence off-targeting within *StALS2* using the gRNA746 reagent, even with mismatches within the PAM core recognition site, highlights the importance of testing multiple sgRNAs before applying CRISPR/Cas towards precise genome editing (Fig 2 and S5 Fig) [28]. Complete mutagenesis of all *StALS* alleles was not observed in the nine primary events analyzed and is most likely due to *ALS* being an essential gene [29]. The maintenance of a single mutation type across clonal generations in the tetraploid background suggests the mutation can be carried into future clonal generations.

**Fig 3:**
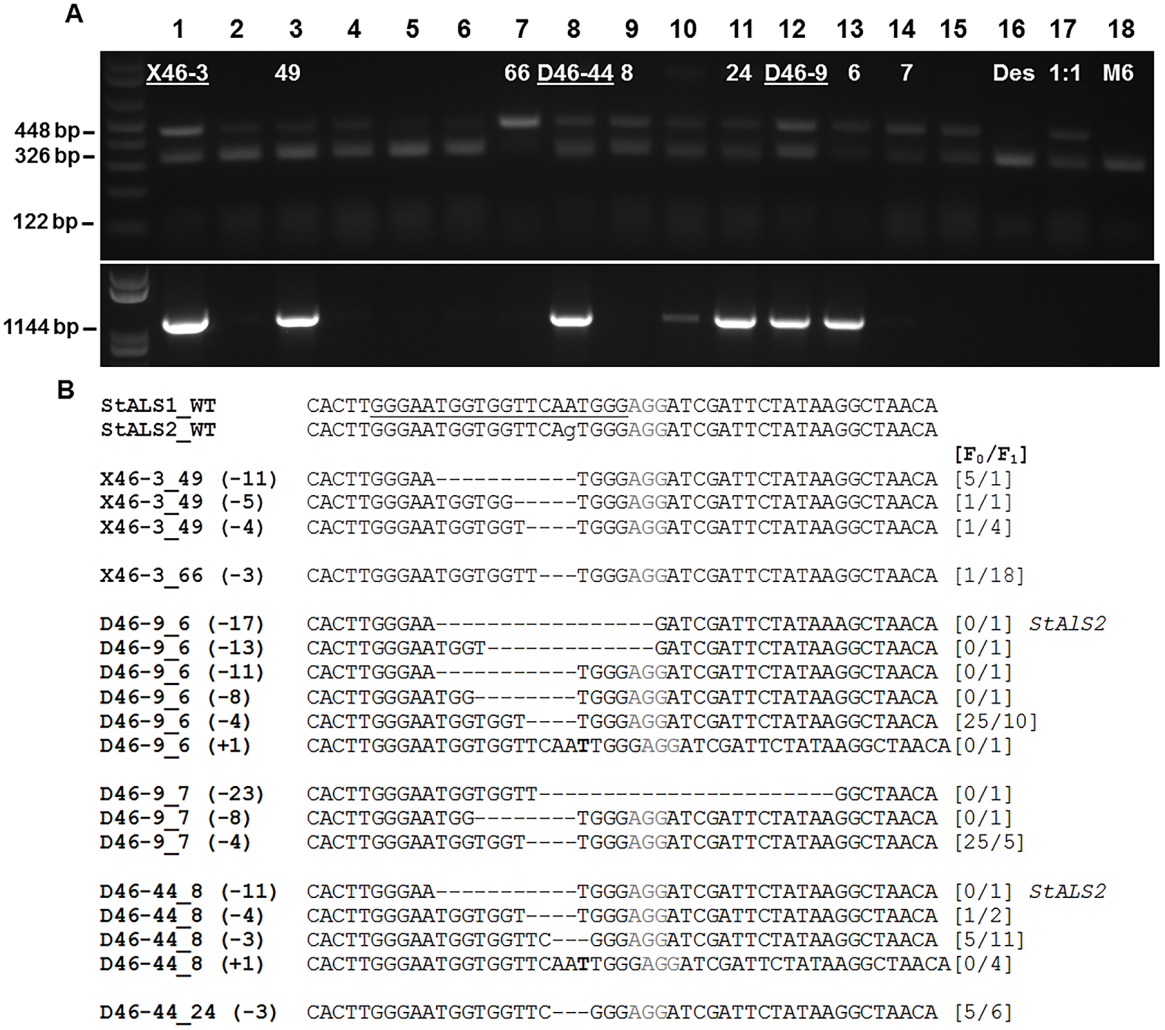
Inheritance of targeted mutations and Cas9 in progeny of primary CRISPR/Cas events. Three primary events with cloned targeted mutations (lanes 1, 8 and 12; underlined) were used to generate genetic populations to assess inheritance of targeted mutations. The diploid event (lane 1; X46-3) was crossed to an inbred diploid line, M6 (lane 18) as the female parent while tetraploid events (lanes 8, 12; D46-44, D46-9) were selfed. Six progeny from the X46-3 population (lanes 2–7) and three progeny from the D46-44 (lanes 9–11) and D46-9 (lanes 13–15) populations were assessed for **A**) targeted mutations using a restriction digestion assay (top gel) and inheritance of Cas9 (bottom gel) and used for **B**) cloning targeted mutations using previously described methods (Fig 2 and S5 Fig). The PCR assay used for detecting Cas9 (**A**; bottom gel) produced a 1144 bp amplicon with each lane corresponding to the top gel and is further described in S6 Fig Wild-type Désirée and M6 were used as negative controls (lanes 16 and 18, respectively) and a 1:1 template mixture with wild-type and mutated DNA was used as a positive control (lane 17). The lengths of deletions (-) or insertions (+) of the targeted mutations in progeny (**B**) are in parenthesis to the left of each cloned mutation and the number of reads generated in the primary event (F_0_) or individual progeny (F_1_) are in brackets on the right. All targeted mutations were aligned to wild-type sequence and cloned from *StALS1* unless indicated on the right. PAM sequences are in gray.

Inheritance of germline mutations and CRISPR/Cas reagents was also evaluated in progeny of three mutant events (Fig 3, S6 and S7 Figs, Table 2). Tetraploid mutant events, D46-9 and -44 were selfed and diploid event, X46-3 was crossed to a self-compatible diploid line, M6 [23]. Progeny from each population were screened for inheritance of CRISPR/Cas reagents (“Cas9”) and Cas9-free progeny were assessed for targeted mutations (Fig 3, S6 and S7 Figs). The percentage of Cas9-free progeny ranged from 19–37% across ploidy types and 87–100% Cas9-free progeny contained targeted mutations (S6 and S7 Figs, Table 2). To determine if targeted mutations detected in progeny were inherited from primary events, two progeny from each population were chosen for mutation cloning. Cas9-containing progeny from X46-3 and D46-9 (X46-3_49 and D46-9_6, respectively) contained new somatic mutations along with mutations from primary events (Fig 3B). Conversely, Cas9-free progeny from all three primary events (X46-3_66, D46-9_7, and D46-44_8) inherited mutations from primary events and new germline mutations with an expected number of mutant alleles allowing for at least one wild-type allele. Interesting, targeted mutations that predominated in tetraploid primary events also predominated in progeny regardless of Cas9 inheritance (ex: D46-44_24 (-3)). This is likely due to opportunity for multiple mutant alleles in the tetraploid background and enrichment of mutant alleles through selfing. Furthermore, the lack of a wild-type band signal from X46-3_66 and the presence of only one mutant type (-3) in *StALS1* is intriguing and could possibly be due to an additional mutation allele in *StALS2* that was not identified and the potentially low impact of a single amino acid deletion, respectively.

**Table 2:** Summary of targeted mutation screen of progeny from primary events and inheritance of Cas9. Progeny from diploid (X46-3) and tetraploid (D46-9, D46-44) primary events were screened for inheritance of Cas9 (# of Cas9-free progeny) and Cas9-free progeny were screened for targeted mutations (# of Cas9-free progeny with mutations) (S6 and S7 Figs). Mutation transmission percentages are of Cas9-free progeny with targeted mutations and percent of Cas9-free are of the number of progeny screened. Mutations detected are targeted mutations cloned from primary events (F_0_) and progeny (F_1_).

Primary event	Mutations detected (bp)	# of progeny screened	# of progeny Cas9-free	# of Cas9-free progeny with mutations	Mutation transmission (%)	Cas9-free (%)
X46-3	-2, -3, -4, -5, -6, -11, -12, -17	48	18	16 out of 18	89%	37%
D46-9	+1, -4, -8, -11, -13, -17, -23	31	6	6 out of 6	100%	19%
D46-44	+1, -3, -4, -10, -11	25	8	7 out of 8	87%	32%

This report in potato and other recent reports in tomato and citrus support the use of CRISPR/Cas for targeted mutagenesis in members of the Solanaceae family and vegetatively propagated plant species [30–33]. The ability to make targeted mutations in diploid potato events and introgression of self-compatibility from self-compatible diploid lines, such as M6 provides a never before opportunity to fix targeted mutations and conduct functional genomics in potato [23]. Furthermore, the geminivirus T-DNA used in this study has previously been shown to be effective for promoting HR in tobacco but could also potentially be used for transient expression of genome editing reagents [19]. This approach would be beneficial in tetraploid potato varieties that cannot be used in genetic crosses to remove genome editing reagents. Nevertheless, further analysis of LSL T-DNA integration and inheritance of targeted mutations derived from geminivirus-modified events must be conducted.

## Supporting Information

S1 Fig Schematic for delivering CRISPR/Cas reagents to potato leaf explants and detecting targeted mutations in callus and primary events. (TIF)

S2 Fig Binary T-DNA vector constructs used for expressing CRISPR/Cas reagents and PCR primers used for detecting reagents. A. Conventional 35S T-DNA backbone (pMDC32; [18]) used to express Cas9 and geminivirus Rep/RepA (Rep) coding sequences [19]. Black and gray arrows represent PCR primers used for detecting Rep and Cas9, respectively (Fig 3, S4 and S6 Figs). B. Geminivirus LSL backbone (pLSL; [19]) with *cis*-acting viral elements, long-intergenic region (LIR) and short-intergenic region (SIR) in an L-S-L arrangement with splicing acceptor (SA) and splicing donor (SD) sites flanking the transcribed region. The pLSL T-DNA does not include Rep and requires co-transformation with the Rep T-DNA for efficient replication. Black arrows represent PCR primers used for detecting the LSL backbone (S4 Fig). C. Upon co-transformation of the pLSL T-DNA with the Rep T-DNA, the viral replicon is released and replicated to a high copy number within the plant nucleus. A doubled 35S promoter (2x35S) was used to drive Cas9 and Rep expression with a nopaline synthase transcriptional terminator (NOS-t). Single-guide RNA (sgRNA) expression is driven by an *Arabidopsis* U6 promoter (U6). T-DNAs are delineated by left (LB) and right (RB) borders and contain a selectable hygromycin-resistance marker gene which is excluded from the viral replicon.
(TIF)

S3 Fig Enrichment PCR assay using potato callus tissue transformed with gRNA746 and gRNA751 CRISPR/Cas reagents. Total genomic DNA was digested overnight with *Alo*I (lanes 1, 2, 3, 4, 9, 11) or *Bsl*I (lanes 5, 6, 7, 8, 10, 12), used for PCR amplification of the *StALS* target site, and redigested overnight to generate an enriched amplicon. For gRNA746, an enriched amplicon of 448 bp (black arrow) and digest products of 326 bp and 122 bp (gray arrows) were generated. Diploid (X; lanes 1–2, 5–6, 9–10) and tetraploid (D; lanes 3–4, 7–8, 11–12) genotypes were tested using both sgRNAs in the conventional 35S (M; lanes 1, 3, 5, 7) and geminivirus LSL (L; lanes 2, 4, 6, 8) T-DNA backbones. Wild-type (wt; lanes 9–12) genomic DNA was used as negative controls.
(TIF)

S4 Fig Detection of LSL and Rep T-DNA integration in primary events. A PCR assay was used to detect integration of LSL T-DNA and Rep T-DNA in co-transformed events of diploid (**A**; X914-10) and tetraploid (**B**; Désirée) potato (S2 Table). Primers specific to the LSL T-DNA and Rep T-DNA were used for top and bottom images of each panel, respectively (S2 Fig). Expected amplicons were 635 bp and 451 bp in size for LSL and Rep T-DNA, respectively and were generated using Phusion High-Fidelity DNA Polymerase (NEB, Ipsich, MA) and total genomic DNA from primary event leaf tissue. Lane numbering follows the order of events listed in S2 Table with lanes 1–13 (X914-10) and lanes 1–12 (Désirée) generated using gRNA746 and lane 14 (X914-10) and lane 13 (Désirée) generated using gRNA751. Wild-type (WT) controls are shown for each genetic background.
(TIF)

S5 Fig Additional targeted mutations in primary events of potato using CRISPR/Cas reagents. Cloned mutations from diploid (X) and tetraploid (D) events constitutively expressing gRNA746 (46) CRISPR/Cas reagents are shown. Sanger reads from each event were aligned to *StALS1* and -*2* wild-type sequence (WT) from the gRNA746 target site. The lengths of deletions (-) or insertions (+) are in parenthesis to the left of each cloned mutation and the number of reads generated in the primary event (T_0_) or first clonal generation (CG_1_) are in brackets on the right. All targeted mutations were cloned from *StALS1* unless indicated on the right. PAM sequences are in gray.
(TIF)

S6 Fig Inheritance of Cas9 in progeny of primary events. A PCR assay was used to detect Cas9 in progeny of diploid (**A**; X46-3) and tetraploid (**B** and **C**; D46-9 and D46-44, respectively) primary events (Fig 3 and Table 2). Primers specific to Cas9 and the *Arabidopsis* U6 promoter were used to generate a 1144 bp expected amplicon (S2 Fig; gray arrows). The expected amplicon was generated using GoTaq^®^ Green Master Mix (Promega, Madison, WI) and total genomic DNA from progeny (**A**; lanes 1–48, **B**; lanes 1–31, **C**; lanes 1–25) and primary events (**A**; lane 50, **B**; lane 32, **C**; lane 27). Wild-type (WT) controls are shown for each genetic background and underlined progeny were used for targeted mutation cloning (Fig 3 and Table 2).
(TIF)

S7 Fig Inheritance of targeted mutations in progeny of primary events. A restriction enzyme digestion assay was used to detect targeted mutations in progeny of diploid (**A**; X46-3) and tetraploid (**B** and **C**; D46-9 and D46-44, respectively) primary events as previously described (Fig 3 and Table 2). Primary amplicons were generated from progeny (**A**; lanes 1–18, **B**; lanes 1–6, **C**; lanes 1–8) and primary events (**A**; lane 20, **B**; lane 8, **C**; lane 10). Wild-type (WT) controls are shown for each genetic background. Mutant (Mut) controls were generated using mutant template DNA.
(TIF)

S1 Table Modified enrichment PCR assay band quantification data. Diploid (X914-10) and tetraploid (Désirée) potato leaf explants were transformed with CRISPR/Cas reagents in the conventional 35S T-DNA (35S), geminivirus LSL T-DNA (LSL) or non-transformed controls (none). *Alo*I and *Bsl*I restriction enzymes were used for gRNA746 and 751, respectively. ImageJ was used for band quantification and normalization was done by dividing enriched by primary band intensities. Modified enrichment PCR results were determined as positive (+), negative (-), or non-detectable (ND) if enriched bands have normalized intensities equal or over 0.5, less than 0.5 and equal or more than 0.05, or less then 0.05, respectively.
(DOCX)

S2 Table Restriction enzyme digestion assay band quantification data from primary events expressing CRISPR/Cas reagents. Diploid (X) and tetraploid (D) primary events generated using gRNA746 (46) and gRNA751 (51) CRISPR/Cas reagents were screened using a restriction enzyme digestion assay (Fig 2A). Resistant (448 bp) and digested (326 and 357 bp for gRNA746 and gRNA751, respectively) bands were quantified using ImageJ software. Digested bands were corrected for size by multiplying the digested band intensity by the size ratio of the resistant band by the digested band (Digested band + correction). Targeted mutation frequency percentages were calculated by dividing the resistant band by the sum of both resistant and digested bands and multiplying by 100. Listed events have targeted mutation frequencies over 1% and are organized by transformation. Events with targeted mutation frequencies over thresholds for expected single allele mutation frequencies (25 and 12.5% for X914-10 and Désirée, respectively) are shaded and bolded events were used for cloning targeted mutations (Fig 2 and S5 Fig). Wild-type (WT) controls are shown using restriction enzyme digestion assays for both gRNA746 and gRNA751.
(DOCX)
