# Supplementary figures and images for: Generation and Inheritance of Targeted Mutations in Potato (Solanum tuberosum L.) Using the CRISPR/Cas System
Source: PLoS One. 2015 Dec 14;10(12):e0144591. doi: 10.1371/journal.pone.0144591 (PMC4684367; doi:10.1371/journal.pone.0144591)

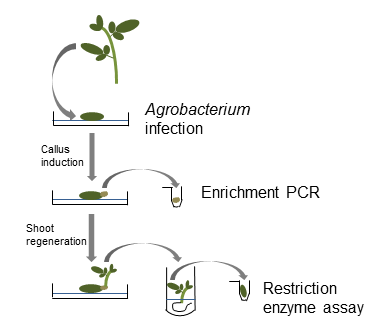

Supplement: S1 Fig — (TIF) [file pone.0144591.s001.tif]

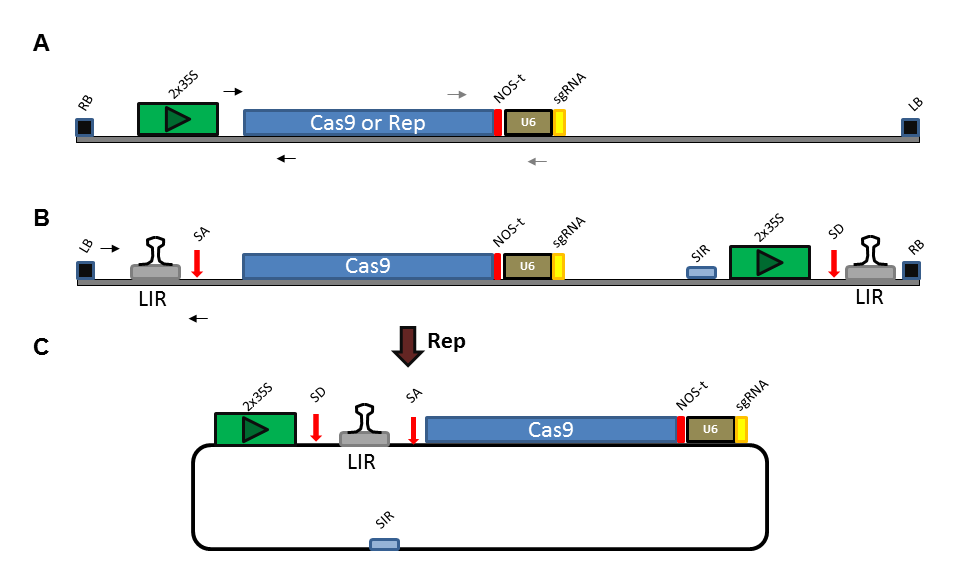

Supplement: S2 Fig — A. Conventional 35S T-DNA backbone (pMDC32; [18]) used to express Cas9 and geminivirus Rep/RepA (Rep) coding sequences [19]. Black and gray arrows represent PCR primers used for detecting Rep and Cas9, respectively (Fig 3, S4 and S6 Figs). B. Geminivirus LSL backbone (pLSL; [19]) with cis-acting viral elements, long-intergenic region (LIR) and short-intergenic region (SIR) in an L-S-L arrangement with splicing acceptor (SA) and splicing donor (SD) sites flanking the transcribed region. The pLSL T-DNA does not include Rep and requires co-transformation with the Rep T-DNA for efficient replication. Black arrows represent PCR primers used for detecting the LSL backbone (S4 Fig). C. Upon co-transformation of the pLSL T-DNA with the Rep T-DNA, the viral replicon is released and replicated to a high copy number within the plant nucleus. A doubled 35S promoter (2x35S) was used to drive Cas9 and Rep expression with a nopaline synthase transcriptional terminator (NOS-t). Single-guide RNA (sgRNA) expression is driven by an Arabidopsis U6 promoter (U6). T-DNAs are delineated by left (LB) and right (RB) borders and contain a selectable hygromycin-resistance marker gene which is excluded from the viral replicon. (TIF) [file pone.0144591.s002.tif]

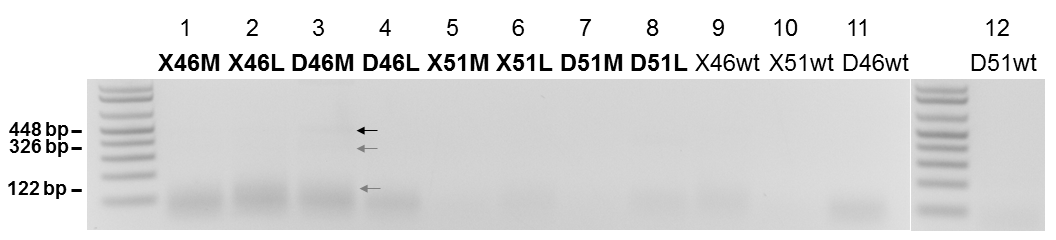

Supplement: S3 Fig — Total genomic DNA was digested overnight with AloI (lanes 1, 2, 3, 4, 9, 11) or BslI (lanes 5, 6, 7, 8, 10, 12), used for PCR amplification of the StALS target site, and redigested overnight to generate an enriched amplicon. For gRNA746, an enriched amplicon of 448 bp (black arrow) and digest products of 326 bp and 122 bp (gray arrows) were generated. Diploid (X; lanes 1–2, 5–6, 9–10) and tetraploid (D; lanes 3–4, 7–8, 11–12) genotypes were tested using both sgRNAs in the conventional 35S (M; lanes 1, 3, 5, 7) and geminivirus LSL (L; lanes 2, 4, 6, 8) T-DNA backbones. Wild-type (wt; lanes 9–12) genomic DNA was used as negative controls. (TIF) [file pone.0144591.s003.tif]

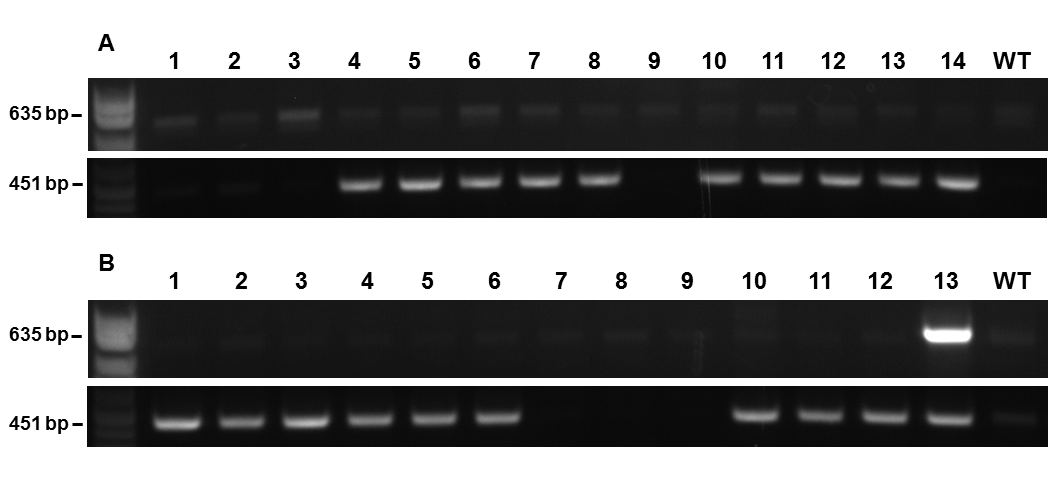

Supplement: S4 Fig — A PCR assay was used to detect integration of LSL T-DNA and Rep T-DNA in co-transformed events of diploid (A; X914-10) and tetraploid (B; Désirée) potato (S2 Table). Primers specific to the LSL T-DNA and Rep T-DNA were used for top and bottom images of each panel, respectively (S2 Fig). Expected amplicons were 635 bp and 451 bp in size for LSL and Rep T-DNA, respectively and were generated using Phusion High-Fidelity DNA Polymerase (NEB, Ipsich, MA) and total genomic DNA from primary event leaf tissue. Lane numbering follows the order of events listed in S2 Table with lanes 1–13 (X914-10) and lanes 1–12 (Désirée) generated using gRNA746 and lane 14 (X914-10) and lane 13 (Désirée) generated using gRNA751. Wild-type (WT) controls are shown for each genetic background. (TIF) [file pone.0144591.s004.tif]

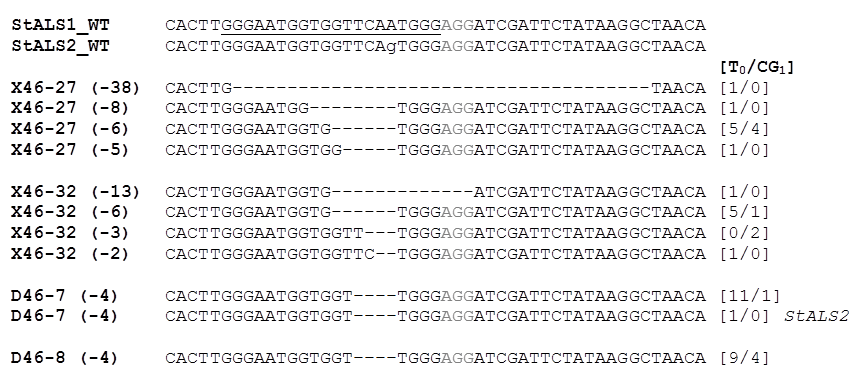

Supplement: S5 Fig — Cloned mutations from diploid (X) and tetraploid (D) events constitutively expressing gRNA746 (46) CRISPR/Cas reagents are shown. Sanger reads from each event were aligned to StALS1 and -2 wild-type sequence (WT) from the gRNA746 target site. The lengths of deletions (-) or insertions (+) are in parenthesis to the left of each cloned mutation and the number of reads generated in the primary event (T0) or first clonal generation (CG1) are in brackets on the right. All targeted mutations were cloned from StALS1 unless indicated on the right. PAM sequences are in gray. (TIF) [file pone.0144591.s005.tif]

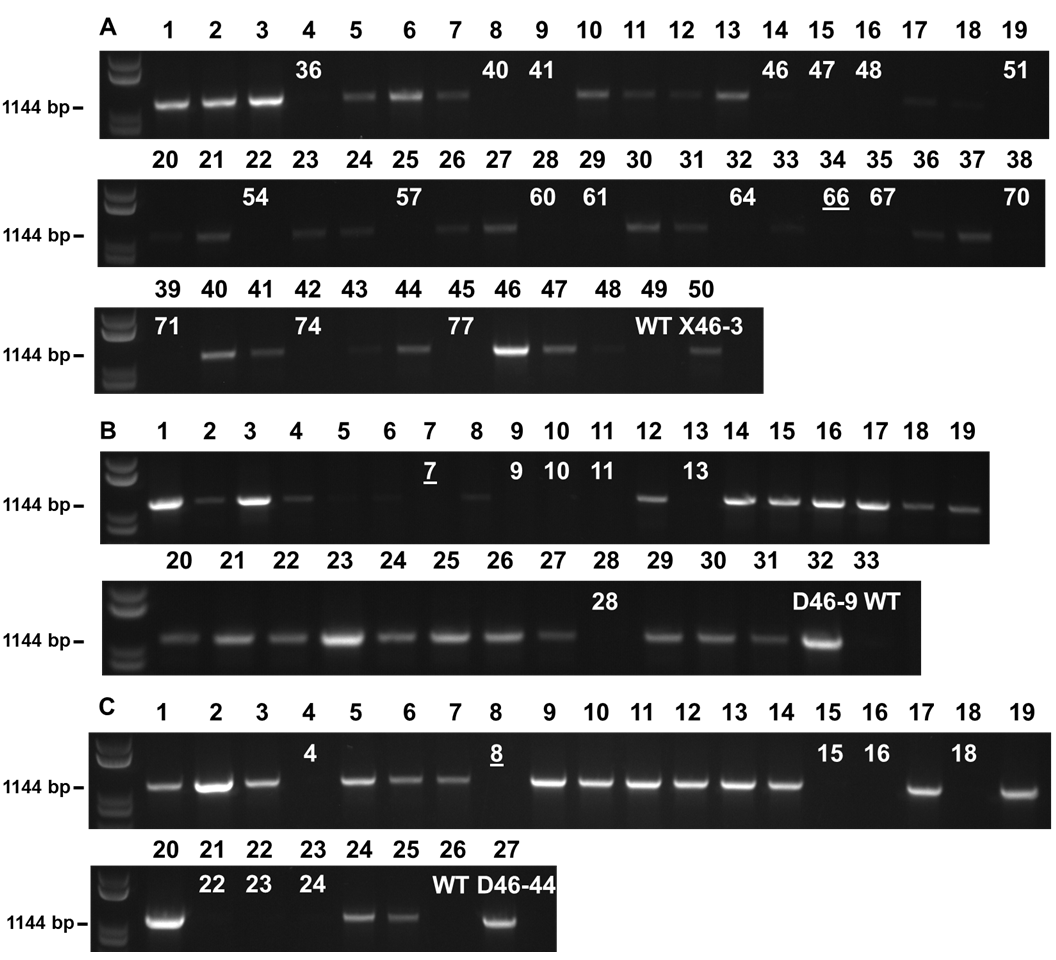

Supplement: S6 Fig — A PCR assay was used to detect Cas9 in progeny of diploid (A; X46-3) and tetraploid (B and C; D46-9 and D46-44, respectively) primary events (Fig 3 and Table 2). Primers specific to Cas9 and the Arabidopsis U6 promoter were used to generate a 1144 bp expected amplicon (S2 Fig; gray arrows). The expected amplicon was generated using GoTaq® Green Master Mix (Promega, Madison, WI) and total genomic DNA from progeny (A; lanes 1–48, B; lanes 1–31, C; lanes 1–25) and primary events (A; lane 50, B; lane 32, C; lane 27). Wild-type (WT) controls are shown for each genetic background and underlined progeny were used for targeted mutation cloning (Fig 3 and Table 2). (TIF) [file pone.0144591.s006.tif]

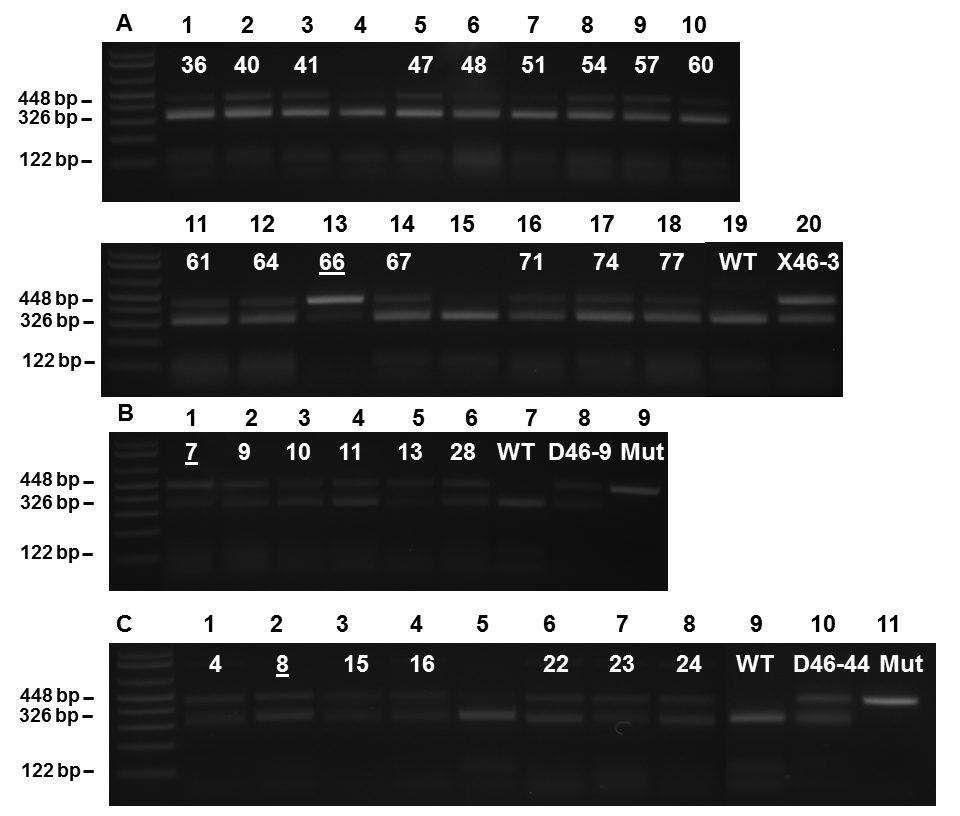

Supplement: S7 Fig — A restriction enzyme digestion assay was used to detect targeted mutations in progeny of diploid (A; X46-3) and tetraploid (B and C; D46-9 and D46-44, respectively) primary events as previously described (Fig 3 and Table 2). Primary amplicons were generated from progeny (A; lanes 1–18, B; lanes 1–6, C; lanes 1–8) and primary events (A; lane 20, B; lane 8, C; lane 10). Wild-type (WT) controls are shown for each genetic background. Mutant (Mut) controls were generated using mutant template DNA. (TIF) [file pone.0144591.s007.tif]
