# Supplementary material for: Generation and Inheritance of Targeted Mutations in Potato (Solanum tuberosum L.) Using the CRISPR/Cas System
Source: PLoS One. 2015 Dec 14;10(12):e0144591. doi: 10.1371/journal.pone.0144591 (PMC4684367; doi:10.1371/journal.pone.0144591)
Supplement: S1 Table — Diploid (X914-10) and tetraploid (Désirée) potato leaf explants were transformed with CRISPR/Cas reagents in the conventional 35S T-DNA (35S), geminivirus LSL T-DNA (LSL) or non-transformed controls (none). AloI and BslI restriction enzymes were used for gRNA746 and 751, respectively. ImageJ was used for band quantification and normalization was done by dividing enriched by primary band intensities. Modified enrichment PCR results were determined as positive (+), negative (-), or non-detectable (ND) if enriched bands have normalized intensities equal or over 0.5, less than 0.5 and equal or more than 0.05, or less then 0.05, respectively. (DOCX) [file pone.0144591.s008.docx]

| **Genotype** | **sgRNA** | **T-DNA** | **Enriched** | **Primary** | **Normalized**  **enriched** | **Modified Enrichment PCR** |
| --- | --- | --- | --- | --- | --- | --- |
| X914-10 | 746 | 35S | 10738.05 | 5024.44 | 2.14 | + |
| X914-10 | 751 | 35S | 4640.20 | 5760.75 | 0.81 | + |
| X914-10 | 746 | LSL | 533.85 | 7090.53 | 0.08 | - |
| X914-10 | 751 | LSL | 773.92 | 11611.19 | 0.07 | - |
| X914-10 | 746 | none | 34.12 | 9487.60 | 0.00 | ND |
| X914-10 | 751 | none | 18.12 | 3230.42 | 0.01 | ND |
| Désirée | 746 | 35S | 13715.77 | 1289.65 | 10.64 | + |
| Désirée | 751 | 35S | 763.51 | 1487.72 | 0.51 | + |
| Désirée | 746 | LSL | 714.68 | 6374.63 | 0.11 | - |
| Désirée | 751 | LSL | 251.78 | 2969.52 | 0.08 | - |
| Désirée | 746 | none | 20.92 | 4384.95 | 0.00 | ND |
| Désirée | 751 | none | 18.53 | 1358.15 | 0.01 | ND |
