# Supplementary material for: Generation and Inheritance of Targeted Mutations in Potato (Solanum tuberosum L.) Using the CRISPR/Cas System
Source: PLoS One. 2015 Dec 14;10(12):e0144591. doi: 10.1371/journal.pone.0144591 (PMC4684367; doi:10.1371/journal.pone.0144591)
Supplement: S2 Table — Diploid (X) and tetraploid (D) primary events generated using gRNA746 (46) and gRNA751 (51) CRISPR/Cas reagents were screened using a restriction enzyme digestion assay (Fig 2A). Resistant (448 bp) and digested (326 and 357 bp for gRNA746 and gRNA751, respectively) bands were quantified using ImageJ software. Digested bands were corrected for size by multiplying the digested band intensity by the size ratio of the resistant band by the digested band (Digested band + correction). Targeted mutation frequency percentages were calculated by dividing the resistant band by the sum of both resistant and digested bands and multiplying by 100. Listed events have targeted mutation frequencies over 1% and are organized by transformation. Events with targeted mutation frequencies over thresholds for expected single allele mutation frequencies (25 and 12.5% for X914-10 and Désirée, respectively) are shaded and bolded events were used for cloning targeted mutations (Fig 2 and S5 Fig). Wild-type (WT) controls are shown using restriction enzyme digestion assays for both gRNA746 and gRNA751. (DOCX) [file pone.0144591.s009.docx]

|  | *Resistant*  *band* | *Digested*  *band* | *Digested*  *band +*  *correction* | *Mutation*  *frequency*  (%) |
| --- | --- | --- | --- | --- |
| X1046M-1 | 102.2 | 6396.9 | 8790.9 | 1.1 |
| **X1046M-3** | **9433.8** | **1471.3** | **2021.9** | **82.3** |
| X1046M-6 | 1928.2 | 5567.0 | 7650.4 | 20.1 |
| X1046M-7 | 640.0 | 3793.5 | 5213.2 | 10.9 |
| X1046M-22 | 1035.5 | 4666.2 | 6412.4 | 13.9 |
| X1046M-24 | 342.9 | 3136.5 | 4310.3 | 7.4 |
| X1046M-25 | 139.4 | 5422.8 | 7452.2 | 1.8 |
| X1046M-26 | 443.7 | 4970.7 | 6830.9 | 6.1 |
| **X1046M-27** | **8974.4** | **2495.9** | **3429.9** | **72.3** |
| X1046M-28 | 833.9 | 5331.4 | 7326.6 | 10.2 |
| X1046M-30 | 731.3 | 1996.6 | 2743.8 | 21.0 |
| X1046M-31 | 2895.5 | 3615.3 | 4968.3 | 36.8 |
| **X1046M-32** | **2635.2** | **2306.9** | **3170.2** | **45.4** |
| X1046M-33 | 122.0 | 6265.9 | 8610.8 | 1.4 |
| X1046M-35 | 95.0 | 5830.4 | 8012.3 | 1.2 |
| X1046L-4 | 213.7 | 11426.6 | 15702.7 | 1.3 |
| X1046L-5 | 1533.9 | 11326.8 | 15565.6 | 9.0 |
| X1046L-6 | 6985.7 | 7020.9 | 9648.3 | 42.0 |
| X1046L-14 | 1645.2 | 9430.7 | 12960.0 | 11.3 |
| X1046L-19 | 530.2 | 3407.1 | 4682.1 | 10.2 |
| X1046L-21 | 137.3 | 3780.8 | 5195.7 | 2.6 |
| X1046L-22 | 668.3 | 5143.7 | 7068.7 | 8.6 |
| X1046L-24 | 159.1 | 9790.1 | 13453.8 | 1.2 |
| X1046L-32 | 2363.5 | 8295.2 | 11399.5 | 17.2 |
| X1046L-33 | 291.7 | 10548.0 | 14495.4 | 2.0 |
| X1046L-36 | 513.4 | 9451.7 | 12988.9 | 3.8 |
| X1046L-37 | 182.8 | 10031.3 | 13785.3 | 1.3 |
| X1046L-38 | 146.8 | 8679.1 | 11927.1 | 1.2 |
| X1051M-15 | 791.5 | 5018.4 | 6297.6 | 11.2 |
| X1051M-18 | 534.8 | 3778.1 | 4741.2 | 10.1 |
| **X1051M-28** | **8957.0** | **3966.9** | **4978.1** | **64.3** |
| X1051L-35 | 54.4 | 2600.4 | 3263.2 | 1.6 |
| Des46M-6 | 316.5 | 4534.8 | 6231.8 | 4.8 |
| **Des46M-7** | **7834.7** | **4335.8** | **5958.4** | **56.8** |
| **Des46M-8** | **8847.1** | **4074.4** | **5599.2** | **61.2** |
| **Des46M-9** | **8357.9** | **3071.7** | **4221.3** | **66.4** |
| Des46M-10 | 159.0 | 4691.1 | 6446.6 | 2.4 |
| Des46M-13 | 644.5 | 5401.9 | 7423.4 | 8.0 |
| Des46M-14 | 715.2 | 5191.2 | 7134.0 | 9.1 |
| Des46M-17 | 1151.2 | 6204.5 | 8526.4 | 11.9 |
| Des46M-22 | 4428.6 | 4547.1 | 6248.7 | 41.5 |
| Des46M-26 | 1120.1 | 5036.9 | 6921.9 | 13.9 |
| Des46M-42 | 420.4 | 5435.1 | 7469.0 | 5.3 |
| Des46M-43 | 1767.6 | 5808.8 | 7982.6 | 18.1 |
| **Des46M-44** | **6278.1** | **1152.1** | **1583.2** | **79.9** |
| Des46M-48 | 78.0 | 5443.6 | 7480.8 | 1.0 |
| Des46M-49 | 1004.6 | 4865.4 | 6686.2 | 13.1 |
| Des46M-51 | 279.7 | 5645.7 | 7758.5 | 3.5 |
| Des46M-52 | 1589.1 | 2840.6 | 3903.6 | 28.9 |
| Des46M-53 | 141.4 | 4739.7 | 6513.4 | 2.1 |
| Des46M-55 | 419.0 | 4767.3 | 6551.4 | 6.0 |
| Des46M-56 | 640.8 | 4016.7 | 5519.9 | 10.4 |
| Des46M-62 | 47553.2 | 6588.9 | 9054.7 | 84.0 |
| Des46L-10 | 284.6 | 5670.9 | 7793.1 | 3.5 |
| Des46L-25 | 495.8 | 6048.8 | 8312.5 | 5.6 |
| Des46L-26 | 646.6 | 8210.9 | 11283.7 | 5.4 |
| Des46L-29 | 1076.8 | 6706.1 | 9215.8 | 10.5 |
| Des46L-30 | 544.6 | 5940.9 | 8164.1 | 6.3 |
| Des46L-31 | 1441.7 | 7718.5 | 10607.1 | 12.0 |
| Des46L-33 | 390.9 | 10328.1 | 14193.2 | 2.7 |
| Des46L-34 | 624.8 | 9424.7 | 12951.7 | 4.6 |
| Des46L-35 | 450.7 | 9086.4 | 12486.9 | 3.5 |
| Des46L-37 | 1151.5 | 10029.9 | 13783.5 | 7.7 |
| Des46L-41 | 359.9 | 9613.3 | 13211.0 | 2.7 |
| Des46L-44 | 261.3 | 6937.9 | 9534.2 | 2.7 |
| **Des51M-5** | **3623.0** | **3276.4** | **4111.6** | **46.8** |
| Des51M-24 | 299.8 | 2938.8 | 3687.9 | 7.5 |
| Des51M-25 | 36.2 | 2335.8 | 2931.2 | 1.2 |
| Des51M-54 | 41.0 | 2611.6 | 3277.2 | 1.2 |
| Des51L-19 | 52.4 | 1411.3 | 1771.1 | 2.9 |
| X914-10 (46) | 4.1 | 5063.3 | 6958.2 | 0.1 |
| Désirée (46) | 10.9 | 12825.1 | 17624.7 | 0.1 |
| X914-10 (51) | 17.3 | 4826.7 | 6057.0 | 0.3 |
| Désirée (51) | 6.1 | 2780.5 | 3489.2 | 0.2 |
